# Hexakis(tetra­aqua­sodium) deca­vanadate(V) dihydrate

**DOI:** 10.1107/S1600536810010251

**Published:** 2010-03-24

**Authors:** Jessica Nilsson, Ebbe Nordlander, Ulrich Behrens, Dieter Rehder

**Affiliations:** a Inorganic Chemistry Research Group, Chemical Physics, Center for Chemistry and Chemical Engineering, Lund University, SE-22100 Lund, Sweden; b Chemistry Department, University of Hamburg, D-20146 Hamburg, Germany

## Abstract

The title compound, {[Na(H_2_O)_4_]_6_[V_10_O_28_]·2H_2_O}_*n*_, crystallized from a H_2_O/THF/CH_3_CN solution (pH *ca* 6) containing equimolar amounts of NaVO_3_ and *N*-(2-hydroxy­benz­yl)-*N*-(2-picol­yl)glycine. In the crystal structure, the deca­vanadate [V_10_O_28_]^6−^ anion (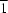 symmetry)  is coordinated, *via* four terminal oxide ligands of V centres, to two dinuclear [{Na(H_2_O)_3_}_2_(μ-H_2_O)_2_]^2+^ units. Inter­connection of these aquasodium-ion-sandwiched deca­vanadates to chains parallel to [001] is effected by μ-[{Na(H_2_O)_3_}_2_(μ-H_2_O)_2_]^2+^ units, bridging adjacent deca­vanadates *via* O=V. The structure is consolidated by an extensive network of  O—H⋯O hydrogen bonds.

## Related literature

Decavanadates with hydrated inorganic cations (Na^+^, K^+^), though with different mol­ecular and supra­molecular arrangements from that in the title structure, have been reported by, for example, Durif *et al.* (1980 ▶); Matias *et al.* (2000 ▶); Lee & Joo (2003 ▶); Wang *et al.* (2003 ▶); Guo & Yao (2007 ▶). More common are deca- and other polyoxido­vanadates with organic counter-ions such as glycyl-glycinium (Crans *et al.*, 1994 ▶) or cryptands and related macrocyclic O_*x*_N_2_ cations (Farahbakhsh *et al.*, 1998 ▶; Wang *et al.*, 2003 ▶). For the impact of deca­vanadates as building blocks for supermolecular assemblies, see: Ferreira da Silva *et al.* (2003 ▶). For the inter­action of deca­vanadate with reverse micelles, see: Baruah *et al.* (2006 ▶).
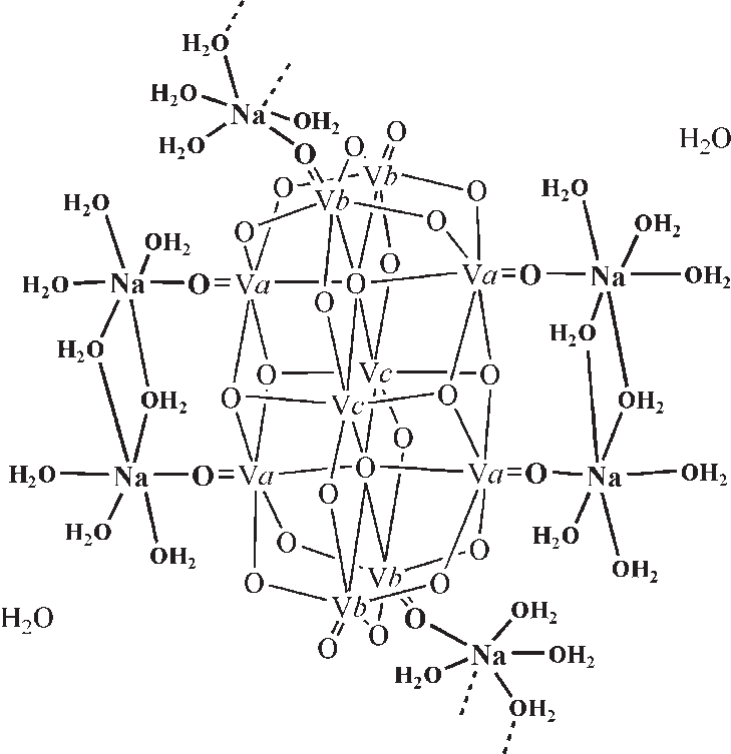


## Experimental

### 

#### Crystal data

[Na(H_2_O)_4_]_6_[V_10_O_28_]·2H_2_O
*M* *_r_* = 1563.76
Triclinic, 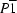

*a* = 9.6144 (9) Å
*b* = 11.7260 (11) Å
*c* = 11.8691 (11) Å
α = 92.446 (1)°
β = 113.582 (1)°
γ = 100.274 (1)°
*V* = 1197.01 (19) Å^3^
*Z* = 1
Mo *K*α radiation
μ = 2.05 mm^−1^
*T* = 100 K
0.50 × 0.24 × 0.10 mm

#### Data collection

Bruker SMART diffractometer
Absorption correction: multi-scan (*SADABS*; Bruker, 2005 ▶) *T*_min_ = 0.428, *T*_max_ = 0.822
7584 measured reflections
5049 independent reflections
4621 reflections with *I* > 2σ(*I*)
*R*_int_ = 0.010

#### Refinement

*R*[*F* ^2^ > 2σ(*F* ^2^)] = 0.022
*wR*(*F* ^2^) = 0.057
*S* = 1.06
5049 reflections
396 parameters
39 restraints
H-atom parameters constrained
Δρ_max_ = 0.36 e Å^−3^
Δρ_min_ = −0.42 e Å^−3^

### 

Data collection: *SMART* (Bruker, 2005 ▶); cell refinement: *SAINT* (Bruker, 2005 ▶); data reduction: *SAINT*; program(s) used to solve structure: *SHELXS97* (Sheldrick, 2008 ▶); program(s) used to refine structure: *SHELXL97* (Sheldrick, 2008 ▶); molecular graphics: *SHELXTL* (Sheldrick, 2008 ▶); software used to prepare material for publication: *SHELXTL*.

## Supplementary Material

Crystal structure: contains datablocks I, global. DOI: 10.1107/S1600536810010251/br2141sup1.cif

Structure factors: contains datablocks I. DOI: 10.1107/S1600536810010251/br2141Isup2.hkl

Additional supplementary materials:  crystallographic information; 3D view; checkCIF report

## Figures and Tables

**Table 1:** Hydrogen-bond geometry (Å, °)

*D*—H⋯*A*	*D*—H	H⋯*A*	*D*⋯*A*	*D*—H⋯*A*
O15—H15*B*⋯O19^i^	0.84 (2)	2.04 (2)	2.862 (2)	169 (3)
O16—H16*B*⋯O18^ii^	0.84 (2)	1.99 (2)	2.820 (2)	171 (2)
O17—H17*A*⋯O13^ii^	0.84 (2)	1.99 (2)	2.816 (2)	166 (3)
O17—H17*B*⋯O10^i^	0.84 (2)	2.01 (2)	2.837 (2)	168 (3)
O18—H18*A*⋯O4^iii^	0.84 (3)	1.98 (3)	2.815 (2)	176 (2)
O18—H18*B*⋯O22^iv^	0.84 (2)	2.01 (2)	2.822 (2)	164 (2)
O19—H19*A*⋯O5^i^	0.84 (2)	1.97 (2)	2.804 (2)	175 (2)
O20—H20*A*⋯O14^v^	0.84 (2)	2.02 (2)	2.846 (2)	170 (2)
O20—H20*B*⋯O6^vi^	0.84 (2)	1.91 (2)	2.724 (2)	164 (2)
O22—H22*A*⋯O27^v^	0.84 (2)	1.93 (2)	2.752 (2)	168 (2)
O23—H23*B*⋯O7^vii^	0.84 (2)	1.86 (2)	2.694 (2)	173 (2)
O25—H25*A*⋯O23^viii^	0.84 (2)	1.88 (2)	2.706 (2)	170 (3)
O25—H25*B*⋯O27^ix^	0.84 (2)	1.99 (2)	2.810 (3)	167 (2)
O26—H26*A*⋯O21^viii^	0.84 (2)	1.97 (2)	2.796 (2)	167 (3)

Symmetry codes: (i) 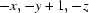; (ii) 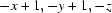; (iii) 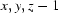; (iv) 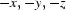; (v) 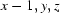; (vi) 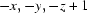; (vii) 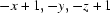; (viii) 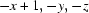; (ix) 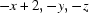.

**Fig. 1.**
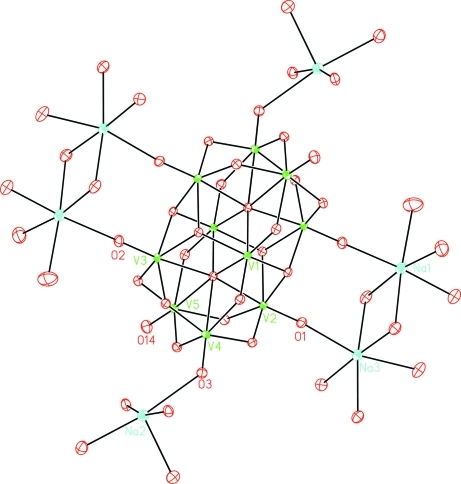
An ORTEP view (50% probability level; hydrogen atoms omitted) of the decavanadate(6-) anion with its sandwiching and bridging [{Na(H2O)3}2(µ-H2O)2]2+ counter-ions.

**Fig. 2.**
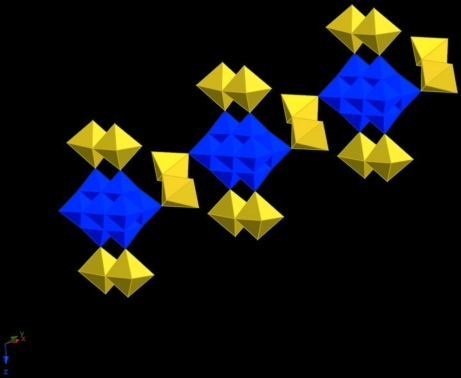
Polygonal representation of a three-membered chain section. The decavanadates are shown in blue, the [{Na(H2O)3}2(µ-H2O)2]2+ counter-ions in yellow.

**Fig. 3.**
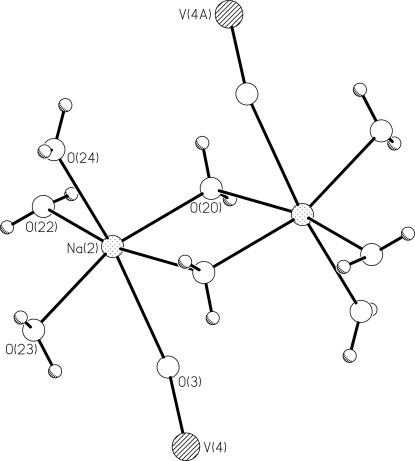
Detail view of the µ-[{Na(H2O)3}2(µ-H2O)2]2+ unit connecting two decavanadates via O4 and O4a.

## supplementary crystallographic information

### Comment

Decavanadates H_n_V_10_O_28_^(6-n)-^ (n = 0-3) form in aqueous solutions containing vanadates (mainly mono- and diprotonated mono- and divanadate, cyclic tetra- and pentavanadate) at a pH < 6.3 and sufficiently high concentrations (> ca. 1 mM, depending on the ionic strength of the medium). In the pH range 4.5 to 6.3, HV_10_O_28_^5-^ is the major species, coexistent with minor amounts of V_10_O_28_^6-^. Stabilisation by organic counter-ions can extend the range of stability of decavanadates into the alkaline regime (Wang *et al.*, 2003). The centro-symmetric decavanadate contains three distinct types of vanadium centres: The eight peripheral vanadium ions, V*a* [O=V(µ-O)_2_(µ_3_-O)_2_(µ_6_-O)] and V*b* [O=V(µ-O)_4_(µ_6_-O)] carry one terminal and five bridging oxido ligands, while the two central V*c* type vanadium ions are hexa-coordinated by bridging oxygens.

The title compound shows a unique interaction between the decavanadate and dinuclear aquasodium counterions: The structure comprises a discrete non-protonated decavanadate anion [V_10_O_28_]^6-^, sandwiched by two [{Na(H_2_O)_3_}_2_(µ-H_2_O)_2_]^2+^ cations via aqua ligands, and further connected to chains by bridging [{Na(H_2_O)_3_}_2_(µ-H_2_O)_2_]^2+^ cations (Figs. 1, 2 and 3). The sandwiching dinuclear aquasodium cations coordinate to the oxido ligands O1 and O2 of the four V*a* type vanadium ions (V2 and V3) of the decavanadate, while two opposed O=V*b* groups (O3=V4) participate in the chain construction; the second O=V*b* group, O14=V5, is not involved in direct bonding. The oxygen O14 is, however, hydrogen bonded to the aqua ligand H20a (O14···H20a = 2.017 Å). In addition, there are two molecules of water of hydration (O27) which are hydrogen bonded to one of the aqua ligands (H27b···O25 = 1.912 Å) and weakly hydrogen bonded to the doubly bridging O9 of decavanadate (H27a···O9 = 2.197 Å).

### Experimental

NaVO_3_ (0.07 g, 0.55 mmol) and *N*-(2-hydroxibenzyl)-N-(2-picolyl)-glycine (H_2_L; 0.15 g, 0.55 mmol) were dissolved in 10 ml of deionised water/THF 9:1 and stirred at room temp. for three hours. The ^51^V NMR showed the presence of H_2_VO_4_^-^, H_2_V_2_O_7_^2-^, V_4_O_12_^4-^, V_5_O_15_^5-^ and an oxidovanadium complex of L^2-^. The solvent volume was reduced to 3 ml, layered with CH_3_CN, and kept at 8 °C. Yellow crystals of the title compound formed within a week.

### Refinement

The positions of the H atoms were taken from the difference Fourier map and refined with H—O distances of 0.84 Å and H—O—H angles of104.5 °.

### Crystal data

**Table:**

[Na(H_2_O)_4_]_6_[V_10_O_28_]·2H_2_O	*Z* = 1
*M**_r_* = 1563.76	*F*(000) = 780
Triclinic, *P*1	*D*_x_ = 2.169 Mg m^−^^3^
Hall symbol: -P 1	Mo *K*α radiation, λ = 0.71073 Å
*a* = 9.6144 (9) Å	Cell parameters from 4566 reflections
*b* = 11.7260 (11) Å	θ = 4.7–55.9°
*c* = 11.8691 (11) Å	µ = 2.05 mm^−^^1^
α = 92.446 (1)°	*T* = 100 K
β = 113.582 (1)°	Block, orange
γ = 100.274 (1)°	0.50 × 0.24 × 0.10 mm
*V* = 1197.01 (19) Å^3^	

### Data collection

**Table:**

Bruker SMART diffractometer	5049 independent reflections
Radiation source: fine-focus sealed tube	4621 reflections with *I* > 2σ(*I*)
graphite	*R*_int_ = 0.010
ω–scan	θ_max_ = 27.5°, θ_min_ = 2.8°
Absorption correction: multi-scan (*SADABS*; Bruker, 2005)	*h* = −11→12
*T*_min_ = 0.428, *T*_max_ = 0.822	*k* = −14→15
7584 measured reflections	*l* = −15→8

### Refinement

**Table:**

Refinement on *F*^2^	Secondary atom site location: difference Fourier map
Least-squares matrix: full	Hydrogen site location: difference Fourier map
*R*[*F*^2^ > 2σ(*F*^2^)] = 0.022	H-atom parameters constrained
*wR*(*F*^2^) = 0.057	*w* = 1/[σ^2^(*F*_o_^2^) + (0.0224*P*)^2^ + 1.1216*P*] where *P* = (*F*_o_^2^ + 2*F*_c_^2^)/3
*S* = 1.06	(Δ/σ)_max_ = 0.001
5049 reflections	Δρ_max_ = 0.36 e Å^−^^3^
396 parameters	Δρ_min_ = −0.42 e Å^−^^3^
39 restraints	Extinction correction: *SHELXL97* (Sheldrick, 2008), Fc^*^=kFc[1+0.001xFc^2^λ^3^/sin(2θ)]^-1/4^
Primary atom site location: structure-invariant direct methods	Extinction coefficient: 0.0004 (2)

### Special details

**Table:**

Experimental. Crystals were grown from a solution of sodiumvanadate in water-THF-methanol
Geometry. All esds (except the esd in the dihedral angle between two l.s. planes) are estimated using the full covariance matrix. The cell esds are taken into account individually in the estimation of esds in distances, angles and torsion angles; correlations between esds in cell parameters are only used when they are defined by crystal symmetry. An approximate (isotropic) treatment of cell esds is used for estimating esds involving l.s. planes.
Refinement. Refinement of *F*^2^ against ALL reflections. The weighted *R*-factor wR and goodness of fit *S* are based on *F*^2^, conventional *R*-factors *R* are based on *F*, with *F* set to zero for negative *F*^2^. The threshold expression of *F*^2^ > σ(*F*^2^) is used only for calculating *R*-factors(gt) etc. and is not relevant to the choice of reflections for refinement. *R*-factors based on *F*^2^ are statistically about twice as large as those based on *F*, and *R*- factors based on ALL data will be even larger. O—H lengths were fixed to 0.84 A, H—O—H angles to 104.5 degrees.

### Fractional atomic coordinates and isotropic or equivalent isotropic displacement parameters (Å^2^)

**Table:**

	*x*	*y*	*z*	*U*_iso_*/*U*_eq_	
V1	0.32866 (3)	0.49894 (2)	0.49773 (3)	0.00662 (7)	
V2	0.36691 (3)	0.43084 (2)	0.25206 (3)	0.00734 (7)	
V3	0.52081 (3)	0.31293 (2)	0.62715 (3)	0.00707 (7)	
V4	0.22632 (3)	0.24259 (2)	0.37700 (3)	0.00789 (7)	
V5	0.54694 (3)	0.24381 (3)	0.38087 (3)	0.00819 (7)	
Na1	0.68380 (9)	0.20347 (7)	−0.04171 (7)	0.01458 (16)	
Na2	0.00968 (8)	−0.06905 (6)	0.36995 (7)	0.01258 (15)	
Na3	0.20510 (8)	0.51845 (6)	−0.09634 (7)	0.01183 (15)	
O1	0.30955 (15)	0.47278 (11)	0.11639 (11)	0.0112 (3)	
O2	0.57790 (15)	0.27004 (11)	0.76214 (12)	0.0117 (3)	
O3	0.06723 (14)	0.14613 (11)	0.33464 (12)	0.0116 (3)	
O4	0.43595 (14)	0.44985 (10)	0.65664 (11)	0.0074 (2)	
O5	0.30505 (14)	0.54867 (10)	0.34087 (11)	0.0078 (2)	
O6	0.32409 (14)	0.22657 (10)	0.54530 (11)	0.0089 (2)	
O7	0.60556 (14)	0.22842 (10)	0.54878 (11)	0.0089 (2)	
O8	0.47848 (14)	0.32382 (10)	0.24227 (11)	0.0091 (2)	
O9	0.19622 (14)	0.32182 (10)	0.23905 (11)	0.0091 (2)	
O10	0.17201 (14)	0.38447 (10)	0.44432 (11)	0.0092 (2)	
O11	0.35146 (14)	0.16030 (10)	0.34055 (11)	0.0093 (2)	
O12	0.45138 (13)	0.38945 (10)	0.44775 (11)	0.0072 (2)	
O13	0.72840 (14)	0.38759 (11)	0.45042 (11)	0.0091 (2)	
O14	0.63150 (15)	0.15212 (11)	0.34249 (12)	0.0134 (3)	
O15	0.08001 (15)	0.64704 (12)	−0.03043 (12)	0.0139 (3)	
O16	0.43615 (15)	0.66748 (12)	−0.03469 (12)	0.0136 (3)	
O17	0.13702 (16)	0.61325 (13)	−0.27889 (12)	0.0163 (3)	
O18	0.31590 (15)	0.38521 (11)	−0.16898 (12)	0.0128 (3)	
O19	−0.03436 (15)	0.37867 (12)	−0.19183 (12)	0.0139 (3)	
O20	−0.18096 (15)	−0.01604 (11)	0.42273 (12)	0.0117 (3)	
O21	0.76870 (16)	0.15411 (13)	0.15731 (13)	0.0178 (3)	
O22	−0.20085 (16)	−0.14460 (12)	0.17792 (13)	0.0178 (3)	
O23	0.23048 (15)	−0.07292 (11)	0.33278 (12)	0.0128 (3)	
O24	0.01105 (16)	−0.26635 (12)	0.41579 (13)	0.0163 (3)	
O25	0.83366 (19)	0.08376 (13)	−0.08832 (14)	0.0236 (3)	
O26	0.47074 (19)	0.03597 (14)	−0.12596 (16)	0.0294 (4)	
O27	0.8411 (2)	−0.14147 (13)	−0.03821 (14)	0.0281 (4)	
H15A	0.0004 (18)	0.657 (2)	−0.0896 (15)	0.0406 (17)*	
H15B	0.055 (3)	0.642 (2)	0.0294 (14)	0.0406 (17)*	
H16A	0.459 (3)	0.670 (2)	−0.0956 (14)	0.0406 (17)*	
H16B	0.509 (2)	0.646 (2)	0.0210 (16)	0.0406 (17)*	
H17A	0.178 (2)	0.601 (3)	−0.3279 (17)	0.0406 (17)*	
H17B	0.0421 (8)	0.605 (3)	−0.3240 (18)	0.0406 (17)*	
H18A	0.349 (3)	0.402 (2)	−0.2226 (19)	0.0406 (17)*	
H18B	0.273 (3)	0.3144 (7)	−0.187 (2)	0.0406 (17)*	
H19A	−0.112 (2)	0.404 (2)	−0.2358 (18)	0.0406 (17)*	
H19B	−0.020 (3)	0.334 (2)	−0.2412 (17)	0.0406 (17)*	
H20A	−0.238 (2)	0.0307 (16)	0.391 (2)	0.0406 (17)*	
H20B	−0.241 (2)	−0.0771 (13)	0.423 (3)	0.0406 (17)*	
H21A	0.8521 (17)	0.131 (2)	0.191 (2)	0.0406 (17)*	
H21B	0.785 (3)	0.2171 (14)	0.201 (2)	0.0406 (17)*	
H22A	−0.202 (3)	−0.148 (2)	0.1070 (11)	0.0406 (17)*	
H22B	−0.279 (2)	−0.118 (2)	0.170 (2)	0.0406 (17)*	
H23A	0.287 (2)	−0.0058 (9)	0.351 (2)	0.0406 (17)*	
H23B	0.287 (2)	−0.1167 (16)	0.373 (2)	0.0406 (17)*	
H24A	0.0900 (17)	−0.291 (2)	0.460 (2)	0.0406 (17)*	
H24B	−0.0612 (19)	−0.304 (2)	0.431 (2)	0.0406 (17)*	
H25A	0.824 (3)	0.085 (2)	−0.1616 (8)	0.0406 (17)*	
H25B	0.9284 (10)	0.109 (2)	−0.0442 (18)	0.0406 (17)*	
H26A	0.410 (3)	−0.0259 (12)	−0.128 (3)	0.0406 (17)*	
H26B	0.519 (3)	0.015 (2)	−0.166 (2)	0.0406 (17)*	
H27A	0.804 (3)	−0.1880 (16)	−0.1037 (13)	0.0406 (17)*	
H27B	0.827 (3)	−0.0763 (10)	−0.061 (2)	0.0406 (17)*	

### Atomic displacement parameters (Å^2^)

**Table:**

	*U*^11^	*U*^22^	*U*^33^	*U*^12^	*U*^13^	*U*^23^
V1	0.00643 (14)	0.00731 (14)	0.00662 (14)	0.00154 (11)	0.00308 (11)	0.00185 (11)
V2	0.00903 (14)	0.00742 (14)	0.00493 (14)	0.00141 (11)	0.00236 (11)	0.00133 (10)
V3	0.00797 (14)	0.00698 (14)	0.00611 (14)	0.00169 (11)	0.00257 (11)	0.00235 (10)
V4	0.00671 (14)	0.00731 (14)	0.00883 (15)	0.00045 (11)	0.00281 (11)	0.00129 (11)
V5	0.00842 (14)	0.00770 (14)	0.00935 (15)	0.00218 (11)	0.00441 (12)	0.00113 (11)
Na1	0.0139 (4)	0.0171 (4)	0.0115 (4)	0.0026 (3)	0.0042 (3)	0.0041 (3)
Na2	0.0119 (4)	0.0134 (3)	0.0121 (4)	0.0025 (3)	0.0049 (3)	0.0001 (3)
Na3	0.0108 (3)	0.0148 (3)	0.0101 (4)	0.0029 (3)	0.0044 (3)	0.0030 (3)
O1	0.0128 (6)	0.0117 (6)	0.0076 (6)	0.0016 (5)	0.0031 (5)	0.0019 (5)
O2	0.0140 (6)	0.0114 (6)	0.0107 (6)	0.0031 (5)	0.0055 (5)	0.0040 (5)
O3	0.0094 (6)	0.0107 (6)	0.0129 (6)	0.0007 (5)	0.0032 (5)	0.0025 (5)
O4	0.0078 (6)	0.0085 (6)	0.0068 (6)	0.0017 (5)	0.0041 (5)	0.0017 (4)
O5	0.0076 (6)	0.0093 (6)	0.0063 (6)	0.0020 (5)	0.0026 (5)	0.0022 (4)
O6	0.0081 (6)	0.0089 (6)	0.0095 (6)	0.0004 (5)	0.0037 (5)	0.0028 (5)
O7	0.0101 (6)	0.0080 (6)	0.0096 (6)	0.0026 (5)	0.0046 (5)	0.0023 (5)
O8	0.0107 (6)	0.0101 (6)	0.0078 (6)	0.0022 (5)	0.0051 (5)	0.0013 (5)
O9	0.0088 (6)	0.0087 (6)	0.0083 (6)	0.0010 (5)	0.0026 (5)	0.0013 (5)
O10	0.0072 (6)	0.0107 (6)	0.0095 (6)	0.0014 (5)	0.0034 (5)	0.0019 (5)
O11	0.0105 (6)	0.0080 (6)	0.0093 (6)	0.0015 (5)	0.0040 (5)	0.0013 (5)
O12	0.0078 (6)	0.0081 (5)	0.0066 (6)	0.0017 (5)	0.0038 (5)	0.0023 (4)
O13	0.0084 (6)	0.0117 (6)	0.0085 (6)	0.0030 (5)	0.0043 (5)	0.0027 (5)
O14	0.0154 (7)	0.0131 (6)	0.0150 (7)	0.0057 (5)	0.0085 (5)	0.0029 (5)
O15	0.0127 (6)	0.0182 (7)	0.0114 (7)	0.0040 (5)	0.0053 (5)	0.0026 (5)
O16	0.0102 (6)	0.0210 (7)	0.0104 (6)	0.0048 (5)	0.0045 (5)	0.0038 (5)
O17	0.0118 (6)	0.0279 (8)	0.0114 (7)	0.0073 (6)	0.0057 (5)	0.0055 (6)
O18	0.0137 (7)	0.0141 (6)	0.0124 (7)	0.0023 (5)	0.0073 (5)	0.0025 (5)
O19	0.0105 (6)	0.0175 (7)	0.0132 (7)	0.0040 (5)	0.0040 (5)	0.0021 (5)
O20	0.0114 (6)	0.0105 (6)	0.0141 (7)	0.0019 (5)	0.0060 (5)	0.0039 (5)
O21	0.0152 (7)	0.0238 (7)	0.0142 (7)	0.0064 (6)	0.0049 (6)	0.0050 (6)
O22	0.0181 (7)	0.0187 (7)	0.0145 (7)	0.0034 (6)	0.0046 (6)	0.0019 (6)
O23	0.0132 (6)	0.0101 (6)	0.0160 (7)	0.0040 (5)	0.0060 (5)	0.0039 (5)
O24	0.0136 (7)	0.0182 (7)	0.0166 (7)	0.0032 (5)	0.0056 (6)	0.0052 (5)
O25	0.0362 (9)	0.0216 (7)	0.0160 (7)	0.0114 (7)	0.0117 (7)	0.0039 (6)
O26	0.0223 (8)	0.0300 (9)	0.0292 (9)	−0.0075 (7)	0.0096 (7)	−0.0008 (7)
O27	0.0489 (10)	0.0138 (7)	0.0126 (7)	−0.0018 (7)	0.0074 (7)	−0.0008 (6)

### Geometric parameters (Å, °)

**Table:**

V1—O13^i^	1.7061 (12)	V5—O7	1.8682 (13)
V1—O10	1.7071 (12)	V5—O8	1.8722 (13)
V1—O5	1.9130 (12)	V5—O13	2.0609 (13)
V1—O4	1.9237 (12)	V5—O12	2.3278 (12)
V1—O12	2.1042 (12)	V5—V1^i^	3.0935 (5)
V1—O12^i^	2.1094 (12)	Na1—O21	2.3073 (16)
V1—V4	3.0832 (5)	Na1—O2^ii^	2.3682 (14)
V1—V5^i^	3.0935 (5)	Na1—O25	2.3760 (17)
V2—O1	1.6102 (13)	Na1—O16^iii^	2.4051 (15)
V2—O8	1.8184 (12)	Na1—O26	2.4108 (17)
V2—O9	1.8393 (12)	Na1—O15^iii^	2.4205 (16)
V2—O4^i^	2.0014 (12)	Na1—Na3^iii^	3.3703 (11)
V2—O5	2.0110 (12)	Na2—O20	2.3303 (15)
V2—O12	2.2400 (12)	Na2—O23	2.3419 (15)
V2—V3^i^	3.0798 (5)	Na2—O22	2.3608 (16)
V2—V5	3.1090 (4)	Na2—O20^iv^	2.3926 (15)
V2—V4	3.1173 (4)	Na2—O24	2.4003 (15)
V3—O2	1.6078 (13)	Na2—O3	2.5741 (15)
V3—O6	1.8191 (12)	Na2—Na2^iv^	3.5112 (15)
V3—O7	1.8240 (12)	Na3—O18	2.3522 (15)
V3—O4	2.0034 (12)	Na3—O15	2.3730 (15)
V3—O5^i^	2.0126 (12)	Na3—O19	2.3831 (15)
V3—O12	2.2414 (12)	Na3—O17	2.3852 (15)
V3—V2^i^	3.0798 (5)	Na3—O16	2.3937 (16)
V3—V5	3.1181 (5)	Na3—O1	2.4391 (14)
V4—O3	1.6095 (13)	Na3—Na1^iii^	3.3704 (11)
V4—O11	1.8333 (12)	O2—Na1^v^	2.3682 (14)
V4—O9	1.8640 (13)	O4—V2^i^	2.0014 (12)
V4—O6	1.8713 (13)	O5—V3^i^	2.0126 (12)
V4—O10	2.0516 (13)	O12—V1^i^	2.1094 (12)
V4—O12	2.3337 (12)	O13—V1^i^	1.7061 (12)
V4—V5	3.0620 (5)	O15—Na1^iii^	2.4205 (16)
V5—O14	1.6095 (13)	O16—Na1^iii^	2.4051 (15)
V5—O11	1.8177 (13)	O20—Na2^iv^	2.3926 (15)
			
O13^i^—V1—O10	106.37 (6)	O11—V5—V1^i^	125.15 (4)
O13^i^—V1—O5	96.99 (6)	O7—V5—V1^i^	77.89 (4)
O10—V1—O5	97.54 (6)	O8—V5—V1^i^	78.58 (4)
O13^i^—V1—O4	97.07 (6)	O13—V5—V1^i^	31.20 (3)
O10—V1—O4	96.89 (6)	O12—V5—V1^i^	42.97 (3)
O5—V1—O4	156.12 (5)	V4—V5—V1^i^	91.989 (11)
O13^i^—V1—O12	165.75 (6)	O14—V5—V2	135.42 (5)
O10—V1—O12	87.88 (5)	O11—V5—V2	82.17 (4)
O5—V1—O12	80.69 (5)	O7—V5—V2	123.05 (4)
O4—V1—O12	80.93 (5)	O8—V5—V2	32.07 (4)
O13^i^—V1—O12^i^	87.52 (5)	O13—V5—V2	81.70 (3)
O10—V1—O12^i^	166.10 (5)	O12—V5—V2	45.93 (3)
O5—V1—O12^i^	80.97 (5)	V4—V5—V2	60.678 (10)
O4—V1—O12^i^	80.46 (5)	V1^i^—V5—V2	61.445 (11)
O12—V1—O12^i^	78.23 (5)	O14—V5—V3	132.95 (5)
O13^i^—V1—V4	145.10 (4)	O11—V5—V3	81.93 (4)
O10—V1—V4	38.74 (4)	O7—V5—V3	31.95 (4)
O5—V1—V4	89.49 (4)	O8—V5—V3	123.26 (4)
O4—V1—V4	89.95 (4)	O13—V5—V3	81.33 (4)
O12—V1—V4	49.15 (3)	O12—V5—V3	45.82 (3)
O12^i^—V1—V4	127.38 (3)	V4—V5—V3	60.666 (9)
O13^i^—V1—V5^i^	38.74 (4)	V1^i^—V5—V3	61.256 (9)
O10—V1—V5^i^	145.11 (4)	V2—V5—V3	91.526 (12)
O5—V1—V5^i^	89.73 (4)	O21—Na1—O2^ii^	172.30 (6)
O4—V1—V5^i^	89.25 (4)	O21—Na1—O25	90.19 (6)
O12—V1—V5^i^	127.01 (3)	O2^ii^—Na1—O25	97.51 (6)
O12^i^—V1—V5^i^	48.78 (3)	O21—Na1—O16^iii^	83.28 (5)
V4—V1—V5^i^	176.157 (13)	O2^ii^—Na1—O16^iii^	89.04 (5)
O1—V2—O8	102.98 (6)	O25—Na1—O16^iii^	171.59 (6)
O1—V2—O9	102.33 (6)	O21—Na1—O26	93.99 (6)
O8—V2—O9	94.90 (6)	O2^ii^—Na1—O26	86.67 (6)
O1—V2—O4^i^	100.68 (6)	O25—Na1—O26	86.32 (6)
O8—V2—O4^i^	90.06 (5)	O16^iii^—Na1—O26	99.38 (6)
O9—V2—O4^i^	154.69 (5)	O21—Na1—O15^iii^	87.34 (5)
O1—V2—O5	100.31 (6)	O2^ii^—Na1—O15^iii^	93.26 (5)
O8—V2—O5	154.79 (5)	O25—Na1—O15^iii^	84.44 (6)
O9—V2—O5	89.42 (5)	O16^iii^—Na1—O15^iii^	89.94 (5)
O4^i^—V2—O5	76.19 (5)	O26—Na1—O15^iii^	170.67 (6)
O1—V2—O12	174.83 (6)	O21—Na1—Na3^iii^	85.15 (4)
O8—V2—O12	80.85 (5)	O2^ii^—Na1—Na3^iii^	89.87 (4)
O9—V2—O12	80.63 (5)	O25—Na1—Na3^iii^	129.08 (5)
O4^i^—V2—O12	75.68 (5)	O16^iii^—Na1—Na3^iii^	45.25 (4)
O5—V2—O12	75.36 (5)	O26—Na1—Na3^iii^	144.54 (5)
O1—V2—V3^i^	90.43 (5)	O15^iii^—Na1—Na3^iii^	44.75 (4)
O8—V2—V3^i^	129.81 (4)	O20—Na2—O23	165.69 (6)
O9—V2—V3^i^	129.49 (4)	O20—Na2—O22	83.88 (5)
O4^i^—V2—V3^i^	39.76 (3)	O23—Na2—O22	104.40 (6)
O5—V2—V3^i^	40.07 (4)	O20—Na2—O20^iv^	83.96 (5)
O12—V2—V3^i^	84.43 (3)	O23—Na2—O20^iv^	87.70 (5)
O1—V2—V5	135.86 (5)	O22—Na2—O20^iv^	167.80 (6)
O8—V2—V5	33.14 (4)	O20—Na2—O24	104.87 (5)
O9—V2—V5	83.38 (4)	O23—Na2—O24	87.28 (5)
O4^i^—V2—V5	87.43 (4)	O22—Na2—O24	88.00 (5)
O5—V2—V5	123.65 (4)	O20^iv^—Na2—O24	94.22 (5)
O12—V2—V5	48.30 (3)	O20—Na2—O3	84.71 (5)
V3^i^—V2—V5	119.735 (13)	O23—Na2—O3	82.43 (5)
O1—V2—V4	135.06 (5)	O22—Na2—O3	99.54 (5)
O8—V2—V4	83.55 (4)	O20^iv^—Na2—O3	80.26 (5)
O9—V2—V4	32.92 (4)	O24—Na2—O3	168.48 (5)
O4^i^—V2—V4	123.96 (4)	O20—Na2—Na2^iv^	42.66 (4)
O5—V2—V4	86.79 (4)	O23—Na2—Na2^iv^	128.01 (5)
O12—V2—V4	48.31 (3)	O22—Na2—Na2^iv^	126.53 (5)
V3^i^—V2—V4	119.559 (13)	O20^iv^—Na2—Na2^iv^	41.30 (3)
V5—V2—V4	58.915 (11)	O24—Na2—Na2^iv^	102.74 (5)
O2—V3—O6	103.48 (6)	O3—Na2—Na2^iv^	79.83 (4)
O2—V3—O7	102.71 (6)	O18—Na3—O15	176.93 (6)
O6—V3—O7	95.00 (6)	O18—Na3—O19	86.84 (5)
O2—V3—O4	100.39 (6)	O15—Na3—O19	90.19 (5)
O6—V3—O4	89.99 (5)	O18—Na3—O17	93.73 (5)
O7—V3—O4	154.50 (5)	O15—Na3—O17	85.47 (5)
O2—V3—O5^i^	99.91 (6)	O19—Na3—O17	89.84 (5)
O6—V3—O5^i^	154.60 (5)	O18—Na3—O16	91.43 (5)
O7—V3—O5^i^	89.34 (5)	O15—Na3—O16	91.37 (5)
O4—V3—O5^i^	76.10 (5)	O19—Na3—O16	170.38 (6)
O2—V3—O12	174.75 (6)	O17—Na3—O16	80.82 (5)
O6—V3—O12	80.41 (5)	O18—Na3—O1	94.70 (5)
O7—V3—O12	80.28 (5)	O15—Na3—O1	86.72 (5)
O4—V3—O12	75.92 (5)	O19—Na3—O1	102.83 (5)
O5^i^—V3—O12	75.68 (5)	O17—Na3—O1	165.13 (6)
O2—V3—V2^i^	90.00 (5)	O16—Na3—O1	86.74 (5)
O6—V3—V2^i^	129.69 (4)	O18—Na3—Na1^iii^	136.94 (4)
O7—V3—V2^i^	129.37 (4)	O15—Na3—Na1^iii^	45.90 (4)
O4—V3—V2^i^	39.71 (4)	O19—Na3—Na1^iii^	135.65 (4)
O5^i^—V3—V2^i^	40.03 (3)	O17—Na3—Na1^iii^	81.97 (4)
O12—V3—V2^i^	84.78 (3)	O16—Na3—Na1^iii^	45.52 (4)
O2—V3—V5	135.29 (5)	O1—Na3—Na1^iii^	83.50 (4)
O6—V3—V5	83.08 (4)	V2—O1—Na3	174.54 (8)
O7—V3—V5	32.82 (4)	V3—O2—Na1^v^	174.33 (8)
O4—V3—V5	124.04 (4)	V4—O3—Na2	132.53 (7)
O5^i^—V3—V5	87.26 (4)	V1—O4—V2^i^	107.65 (6)
O12—V3—V5	48.14 (3)	V1—O4—V3	107.11 (6)
V2^i^—V3—V5	119.925 (12)	V2^i^—O4—V3	100.53 (5)
O3—V4—O11	102.05 (6)	V1—O5—V2	107.67 (6)
O3—V4—O9	103.67 (6)	V1—O5—V3^i^	107.41 (6)
O11—V4—O9	91.64 (6)	V2—O5—V3^i^	99.89 (5)
O3—V4—O6	101.25 (6)	V3—O6—V4	115.52 (6)
O11—V4—O6	91.17 (6)	V3—O7—V5	115.23 (6)
O9—V4—O6	153.74 (5)	V2—O8—V5	114.79 (6)
O3—V4—O10	101.85 (6)	V2—O9—V4	114.65 (6)
O11—V4—O10	156.05 (5)	V1—O10—V4	109.89 (6)
O9—V4—O10	84.06 (5)	V5—O11—V4	114.00 (6)
O6—V4—O10	82.86 (5)	V1—O12—V1^i^	101.77 (5)
O3—V4—O12	175.93 (6)	V1—O12—V2	93.61 (5)
O11—V4—O12	81.69 (5)	V1^i^—O12—V2	93.48 (5)
O9—V4—O12	77.65 (5)	V1—O12—V3	93.23 (5)
O6—V4—O12	76.94 (5)	V1^i^—O12—V3	93.28 (5)
O10—V4—O12	74.37 (5)	V2—O12—V3	169.21 (6)
O3—V4—V5	134.88 (5)	V1—O12—V5	169.98 (6)
O11—V4—V5	32.84 (4)	V1^i^—O12—V5	88.26 (4)
O9—V4—V5	84.37 (4)	V2—O12—V5	85.77 (4)
O6—V4—V5	83.93 (4)	V3—O12—V5	86.05 (4)
O10—V4—V5	123.22 (4)	V1—O12—V4	87.85 (4)
O12—V4—V5	48.85 (3)	V1^i^—O12—V4	170.38 (6)
O3—V4—V1	133.22 (5)	V2—O12—V4	85.91 (4)
O11—V4—V1	124.69 (4)	V3—O12—V4	86.02 (4)
O9—V4—V1	78.79 (4)	V5—O12—V4	82.12 (4)
O6—V4—V1	78.18 (4)	V1^i^—O13—V5	110.05 (6)
O10—V4—V1	31.37 (3)	Na3—O15—Na1^iii^	89.35 (5)
O12—V4—V1	43.00 (3)	Na3—O15—H15A	111.5 (18)
V5—V4—V1	91.854 (11)	Na1^iii^—O15—H15A	119.1 (19)
O3—V4—V2	135.91 (5)	Na3—O15—H15B	126.4 (18)
O11—V4—V2	81.71 (4)	Na1^iii^—O15—H15B	105.9 (19)
O9—V4—V2	32.43 (4)	Na3—O16—Na1^iii^	89.23 (5)
O6—V4—V2	122.73 (4)	Na3—O16—H16A	106.5 (19)
O10—V4—V2	82.14 (4)	Na1^iii^—O16—H16A	129.7 (17)
O12—V4—V2	45.79 (3)	Na3—O16—H16B	108.6 (19)
V5—V4—V2	60.407 (10)	Na1^iii^—O16—H16B	114.1 (17)
V1—V4—V2	61.458 (11)	Na3—O17—H17A	118.5 (18)
O14—V5—O11	103.91 (6)	Na3—O17—H17B	116.8 (18)
O14—V5—O7	101.10 (6)	Na3—O18—H18A	120.3 (18)
O11—V5—O7	91.89 (6)	Na3—O18—H18B	120.2 (18)
O14—V5—O8	103.41 (6)	Na3—O19—H19A	116.6 (19)
O11—V5—O8	91.81 (6)	Na3—O19—H19B	103.9 (19)
O7—V5—O8	153.53 (5)	Na2—O20—Na2^iv^	96.04 (5)
O14—V5—O13	99.72 (6)	Na2—O20—H20A	128.0 (19)
O11—V5—O13	156.35 (5)	Na2^iv^—O20—H20A	107.5 (19)
O7—V5—O13	82.59 (5)	Na2—O20—H20B	108.1 (18)
O8—V5—O13	83.57 (5)	Na2^iv^—O20—H20B	110.5 (19)
O14—V5—O12	173.77 (6)	Na1—O21—H21A	122 (2)
O11—V5—O12	82.18 (5)	Na1—O21—H21B	103.8 (19)
O7—V5—O12	77.12 (5)	Na2—O22—H22A	128.5 (18)
O8—V5—O12	77.44 (5)	Na2—O22—H22B	111.2 (18)
O13—V5—O12	74.17 (5)	Na2—O23—H23A	109.7 (19)
O14—V5—V4	137.06 (5)	Na2—O23—H23B	115.3 (19)
O11—V5—V4	33.16 (4)	Na2—O24—H24A	125.2 (18)
O7—V5—V4	84.53 (4)	Na2—O24—H24B	119.5 (17)
O8—V5—V4	84.34 (4)	Na1—O25—H25A	111.3 (19)
O13—V5—V4	123.19 (4)	Na1—O25—H25B	110.5 (19)
O12—V5—V4	49.02 (3)	Na1—O26—H26A	159 (2)
O14—V5—V1^i^	130.92 (5)	Na1—O26—H26B	85.0 (17)

Symmetry codes: (i) −*x*+1, −*y*+1, −*z*+1; (ii) *x*, *y*, *z*−1; (iii) −*x*+1, −*y*+1, −*z*; (iv) −*x*, −*y*, −*z*+1; (v) *x*, *y*, *z*+1.

### Hydrogen-bond geometry (Å, °)

**Table:**

*D*—H···*A*	*D*—H	H···*A*	*D*···*A*	*D*—H···*A*
O15—H15A···O9^vi^	0.84 (2)	2.07 (2)	2.912 (2)	178 (2)
O15—H15B···O19^vi^	0.84 (2)	2.04 (2)	2.862 (2)	169 (3)
O16—H16A···O8^iii^	0.84 (2)	2.06 (2)	2.892 (2)	178 (3)
O16—H16B···O18^iii^	0.84 (2)	1.99 (2)	2.820 (2)	171 (2)
O17—H17A···O13^iii^	0.84 (2)	1.99 (2)	2.816 (2)	166 (3)
O17—H17B···O10^vi^	0.84 (2)	2.01 (2)	2.837 (2)	168 (3)
O18—H18A···O4^ii^	0.84 (3)	1.98 (3)	2.815 (2)	176 (2)
O18—H18B···O22^vii^	0.84 (2)	2.01 (2)	2.822 (2)	164 (2)
O19—H19A···O5^vi^	0.84 (2)	1.97 (2)	2.804 (2)	175 (2)
O19—H19B···O24^vii^	0.84 (2)	2.23 (2)	3.022 (2)	158 (2)
O20—H20A···O14^viii^	0.84 (2)	2.02 (2)	2.846 (2)	170 (2)
O20—H20B···O6^iv^	0.84 (2)	1.91 (2)	2.724 (2)	164 (2)
O21—H21A···O3^ix^	0.84 (2)	2.06 (2)	2.821 (2)	150 (2)
O21—H21B···O17^iii^	0.84 (2)	2.03 (2)	2.843 (2)	162 (2)
O22—H22A···O27^viii^	0.84 (2)	1.93 (2)	2.752 (2)	168 (2)
O22—H22B···O26^vii^	0.84 (2)	2.12 (2)	2.950 (3)	170 (2)
O23—H23A···O11	0.84 (2)	1.96 (2)	2.756 (2)	158 (2)
O23—H23B···O7^x^	0.84 (2)	1.86 (2)	2.694 (2)	173 (2)
O24—H24A···O13^x^	0.84 (2)	2.19 (2)	3.016 (2)	168 (2)
O24—H24B···O10^iv^	0.84 (2)	2.29 (2)	3.072 (2)	155 (2)
O25—H25A···O23^xi^	0.84 (2)	1.88 (2)	2.706 (2)	170 (3)
O25—H25B···O27^xii^	0.84 (2)	1.99 (2)	2.810 (3)	167 (2)
O26—H26A···O21^xi^	0.84 (2)	1.97 (2)	2.796 (2)	167 (3)
O26—H26B···O14^xi^	0.84 (3)	2.56 (2)	3.026 (2)	116 (2)
O27—H27A···O9^xi^	0.84 (2)	2.20 (2)	2.983 (2)	156 (3)
O27—H27B···O25	0.84 (2)	1.91 (2)	2.739 (2)	169 (2)

Symmetry codes: (vi) −*x*, −*y*+1, −*z*; (iii) −*x*+1, −*y*+1, −*z*; (ii) *x*, *y*, *z*−1; (vii) −*x*, −*y*, −*z*; (viii) *x*−1, *y*, *z*; (iv) −*x*, −*y*, −*z*+1; (ix) *x*+1, *y*, *z*; (x) −*x*+1, −*y*, −*z*+1; (xi) −*x*+1, −*y*, −*z*; (xii) −*x*+2, −*y*, −*z*.
